# Reentrant Information Flow in Electrophysiological Rat Default Mode Network

**DOI:** 10.3389/fnins.2017.00093

**Published:** 2017-02-27

**Authors:** Wei Jing, Daqing Guo, Yunxiang Zhang, Fengru Guo, Pedro A. Valdés-Sosa, Yang Xia, Dezhong Yao

**Affiliations:** ^1^Key Laboratory for NeuroInformation of Ministry of Education, Center for Information in Medicine, School of Life Science and Technology, University of Electronic Science and Technology of ChinaChengdu, China; ^2^Cuban Neurosciences CenterHavana, Cuba

**Keywords:** directed phase transfer entropy, default mode network, information flow, wakeful rest, sleep, rat

## Abstract

Functional MRI (fMRI) studies have demonstrated that the rodent brain shows a default mode network (DMN) activity similar to that in humans, offering a potential preclinical model both for physiological and pathophysiological studies. However, the neuronal mechanism underlying rodent DMN remains poorly understood. Here, we used electrophysiological data to analyze the power spectrum and estimate the directed phase transfer entropy (dPTE) within rat DMN across three vigilance states: wakeful rest (WR), slow-wave sleep (SWS), and rapid-eye-movement sleep (REMS). We observed decreased gamma powers during SWS compared with WR in most of the DMN regions. Increased gamma powers were found in prelimbic cortex, cingulate cortex, and hippocampus during REMS compared with WR, whereas retrosplenial cortex showed a reverse trend. These changed gamma powers are in line with the local metabolic variation of homologous brain regions in humans. In the analysis of directional interactions, we observed well-organized anterior-to-posterior patterns of information flow in the delta band, while opposite patterns of posterior-to-anterior flow were found in the theta band. These frequency-specific opposite patterns were only observed in WR and REMS. Additionally, most of the information senders in the delta band were also the receivers in the theta band, and vice versa. Our results provide electrophysiological evidence that rat DMN is similar to its human counterpart, and there is a frequency-dependent reentry loop of anterior-posterior information flow within rat DMN, which may offer a mechanism for functional integration, supporting conscious awareness.

## Introduction

The default mode network (DMN) is a set of distributed human brain regions that are characterized by their reduced activities during attention-demanding tasks (Fox et al., 2005; Raichle, 2006; Buckner et al., 2008). Recently, a similar network has also been demonstrated in rodents (Lu et al., 2012; Schwarz et al., 2013; Sforazzini et al., 2014; Gozzi and Schwarz, 2016). Anatomically, the rat DMN includes prelimbic cortex (PrL), orbital cortex (Orb), cingulate cortex (Cg), retrosplenial cortex (RSC), posterior parietal cortex (PPC), secondary visual cortex (V2), hippocampus (Hip), and auditory/temporal association cortex (Au/TeA) (Lu et al., 2012). Functionally, DMN is thought to support spontaneous cognition or monitor the environment (Raichle et al., 2001; Buckner et al., 2008; Mantini and Vanduffel, 2013), both of which can be combined for a function of conscious experience, i.e., integrated internal representations of internal and external milieu (Mantini and Vanduffel, 2013). Dysregulation of DMN has been linked to various neurological and psychiatric disorders (Greicius et al., 2004; Öngür et al., 2010; Luo et al., 2011). The genetic and environmental conditions can be specifically controlled in rodents (Gozzi and Schwarz, 2016) to offer preclinical models that shed light on these physiological and pathophysiological determinants.

Accumulating evidence indicates that sleep modulates activities of distributed brain regions, especially the DMN (Maquet et al., 1996, 1997; Braun et al., 1997; Nofzinger et al., 1997; Maquet, 2000; Fox et al., 2013). In recent years, a growing number of studies have indicated that the functional structure of DMN is also modulated by sleep. For example, compared with that in wakeful rest (WR), functional connectivity between anterior and posterior DMN decreases or even uncouples during slow wave sleep (SWS) (Horovitz et al., 2009; Sämann et al., 2011; Chow et al., 2013). Moreover, increased functional connectivity has also been observed during rapid-eye-movement sleep (REMS) compared with WR (Chow et al., 2013). However, to our knowledge, little is known about the directional interactions within DMN across vigilance states (including WR, SWS, and REMS).

Exploring the neurophysiological basis of the neuronal dynamics is a critical step toward understanding the functional role of brain activity (Arieli et al., 1996; Tsodyks et al., 1999; Mantini et al., 2007). To date, however, almost all of the rodent DMN studies have been based on fMRI (Gozzi and Schwarz, 2016, also see Li et al., 2015), where the method is limited to the temporal resolution and indirect relation to neuronal activity (Ramsey et al., 2010; Webb et al., 2013). In contrast, electrophysiological recording (such as local field potential, LFP) offers a direct measurement and finer temporal resolution of the neuronal activity (Buzsáki and Draguhn, 2004; Buzsáki, 2006), which help clarify the intrinsic activity.

In this study, we collected electrophysiological data of the rat DMN across different vigilance states. Power spectrum analysis and directed phase transfer entropy (dPTE) (Hillebrand et al., 2016) were applied to the time series across three vigilance states. The results showed that the power spectrum of each DMN region changed across vigilance states. Furthermore, DMN showed a frequency-dependent (delta-theta) reciprocal information flow between its anterior-posterior parts, a phenomenon which was only found in WR and REMS states. These results complement the electrophysiological evidence of rat DMN, and might provide meaningful insights into the relationship between consciousness and directed interaction within DMN.

## Materials and methods

### Animals

Sixteen male Wistar rats (Chengdu Dossy Experimental Animals Co., Ltd) were housed in small groups with *ad-libitum* access to water and food before surgery. At the time of the surgery, the body weights of the rats ranged from 260 to 290 g. The animals were caged individually during recovery. Housing conditions were set with a 12-h light/dark cycle (white lights on at 8:00). All experiments were approved by the Ethical Committee on Animal Experimentation of the University of Electronic Science and Technology of China (UESTC).

### Surgery

**Figure 1:**
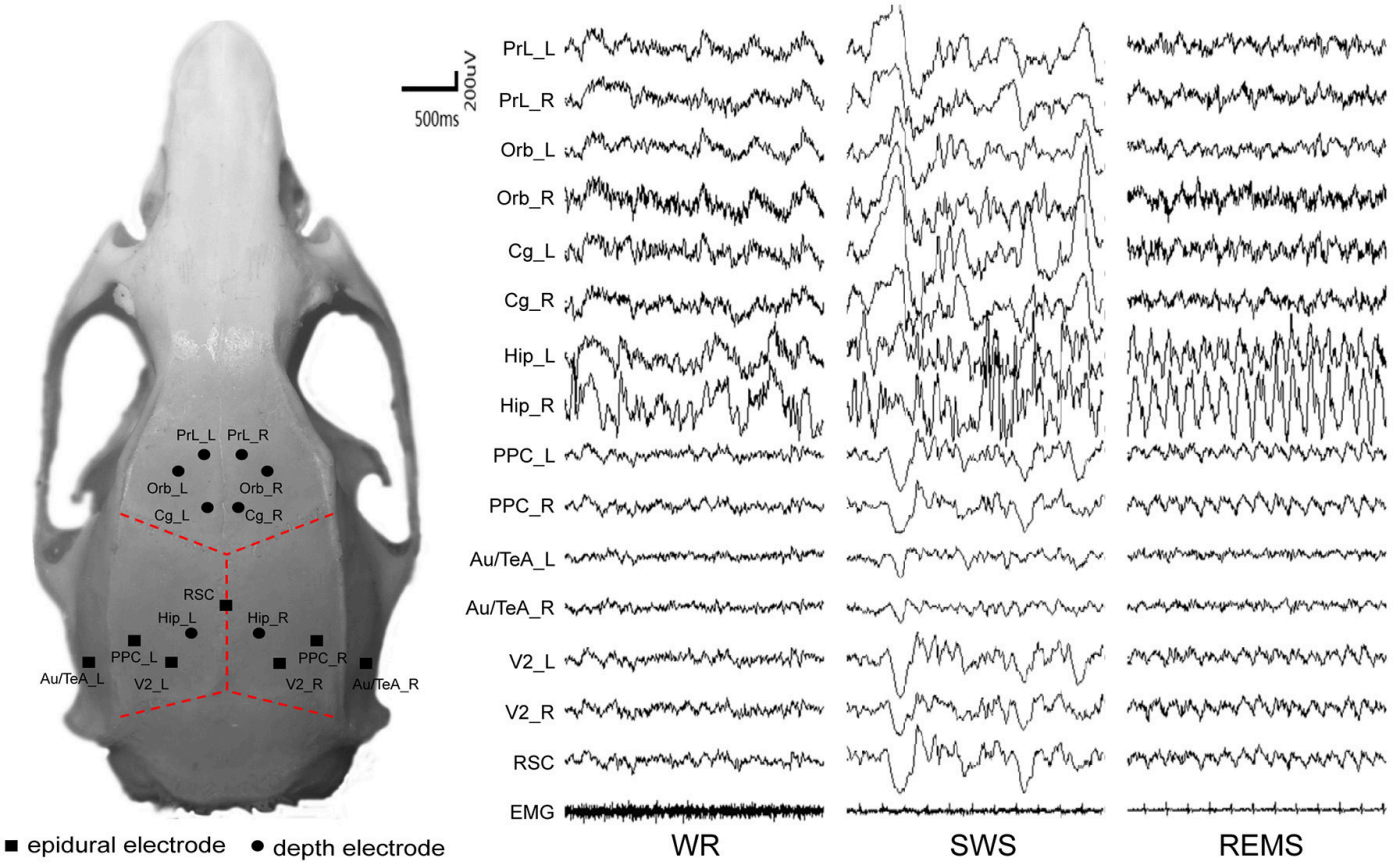
**The distribution of intracranial electrodes and their corresponding EEG tracing**. EMG, electromyography; L, left; R, right. WR, SWS, and REMS are three vigilance states.

Chronic electrode implantation was performed under deep anesthesia (sodium pentobarbital 60 mg/kg body weight, i.p.). To prevent excessive secretion of the respiratory tract, an 0.6 ml of atropine sulfate (0.5 mg/ml, s.c.) was injected. Complementary pentobarbital (15 mg/kg) was given intraperitoneally when required. Local analgesia was administered with lignocaine (2%) for the resection of the temporal muscle. The body heat of the rats was maintained at 37 degrees centigrade with a heating pad. All stereotactic coordinates were relative to bregma according to the atlas (Paxinos and Watson, 2005). Each rat carried fifteen electrodes to record the electroencephalogram (EEG), including seven epidural cortical electrodes and eight depth electrodes according to the coordinates introduced in a fMRI study (Lu et al., 2012). Small holes were drilled in the skull for electrodes. Stainless-steel screw electrodes (diameter, 500 μm) were implanted in the drilled holes as the epidural cortical electrodes; insulated nichrome wires (diameter, 200 μm) were implanted in the drilled holes as the depth electrodes (fixed to the skull with medicinal adhesive). Reference was set at the cerebellum. The coordinates and montage of the electrodes are shown in Table 1 and left inset of Figure 1, respectively. The temporal electrode implantation followed the procedure reported by Meeren et al. (2001). Two electromyographic (EMG) electrodes were implanted bilaterally in the dorsal neck muscles. All the electrodes were welded to connectors and fixed on the skull of the rat with dental acrylic. After the surgical procedure, penicillin G was used for anti-infection, and all rats were given at least 2 weeks to recover before the recording session started.

**Table 1:** **Coordinates of the 15 electrodes**.

**Region**	**Paxino's atlas**		
	**A-P**	**M-L**	**D-V**
PrL	4.2	±0.8	3
Orb	3.7	±1.8	4.7
Cg	1.7	±0.7	2.6
RSC	−3.3	0	0
Hip	−4.3	±1.4	3
PPC	−4.5	±4	0
V2	−5.2	±2.4	0
Au/TeA	−5.2	±8	5

*A-P, M-L, and D-V indicate anterior-posterior, medial-lateral, and dorsal-ventral directions, respectively. PrL, prelimbic cortex; Orb, orbital cortex; Cg, cingulate cortex; RSC, retrosplenial cortex; Hip, hippocampus; PPC, posterior parietal cortex; V2, secondary visual cortex; Au/TeA, Auditory/temporal association cortex*.

### Recording

The rats were habituated to the experimental environment and the recording cable 2 days prior to the recording. Rats were placed in a 40^*^50^*^60 cm glass box after the adaptive procedure. Each recording electrode was connected to an acquisition system (Chengyi, RM62160, China). Electrophysiological signals and videos were synchronously recorded for 72 h (onset at 12:00). The amplified and filtered (0.16–100 Hz for EEG, 8.3–500 Hz for MEG, and 50 Hz notch filter) signals were stored on a hard disk (Lenovo Company, USA). The sample frequency was set at 1,000 Hz. Experiments were performed in a noise-attenuated room, in which the background noise was 32.2 ± 3.0 dB (mean ± SD) and temperature was maintained at 25 ± 0.5 degrees centigrade. The light cycle was set as mentioned above. The experimenter entered the noise-attenuated room to replace food and water and clean cages at 12:00 every day.

### Histology

At the end of the experiment, all animals were deeply anesthetized and perfused intracardially with 0.9% saline followed by a 4% paraformaldehyde phosphate buffered solution. The brains were removed and fixed in the paraformaldehyde phosphate buffer overnight before gradient dehydration with sucrose (15 and 30%). After the brains were adequately fixed, a freezing microtome was used to obtain 30 um coronal sections. The brain sections were stained with ferric-chloride solution on Poly-L-Lysine-coated slides, cover-slipped with DPX mountant, and digitally photographed. After histological inspection, the rats with any electrode tipping out of the designed anatomical location were excluded (6 rats), and the rest 10 rats were included in further analyses (Supplementary Figure 1).

### Data selection

Data were selected from the last 24 h of the recording, and the selection rules of each state were based on EEG, MEG, and behaviors, which are summarized in Table 2 (For more details, see also the SI and right inset of Figure 1).

**Table 2:** **Rules for selection of data**.

**State**	**Electrical activity**		**Behaviors**	**Data size**
	**EEG**	**EMG**		
WR	Low-amplitude, mixed-frequency EEG activity	Relatively low and stable EMG activity	Standing or sitting quietly	300 s, 10 s per segment
SWS	High-amplitude, low-frequency EEG activity	Low-level EMG activity	Lying or curl itself up	300 s, 10 s per segment
REMS	Sawtooth-pattern EEG activity	Flat EMG activity	Lying or curl itself up	300 s, 10 s per segment

### Data analyses

Analyses were performed by using the BrainWave (version 0.9.151.7.2; home.kpn.nl/stam7883/brainwave.html) and our own custom Matlab (release 2013a) scripts.

#### Power spectra analysis

Each 10-s segment of the three states was first band-pass filtered (0.5–46 Hz, excluding potential high-frequency noise, such as muscle activities), down sampled to 256 Hz and de-trended. Then, Welch's method with the Hamming window was used for power spectra calculation in 1 Hz resolution. The log-transformed power spectra of rats, states and brain regions were averaged (one value for each rat, state, and brain region; homologous areas were averaged as one region) for further statistical analysis.

#### Information flow estimation

Both Granger Causality Analysis (GCA) and Dynamic Causal Modeling (DCM) are widely used to measure the directional causal relationship between brain regions (Friston et al., 2013; Seth et al., 2015). However, GCA is based on the linear vector autoregressive (VAR) modeling of signals and their interactions, which needs stationary data (Bressler and Seth, 2011) and is therefore not suitable for non-stationary electrophysiological signals. As for DCM, the parameters are too complicated and the computational cost too high (Lobier et al., 2014) to favor its application in our present data. Thus, we used a recently introduced, model-free, data-driven measure of directional causal relationship, the dPTE (Hillebrand et al., 2016), to estimate the information flow between brain regions of rat DMN. The dPTE is based on information theory, comparing conditional probabilities of phasic information between two signals (Lobier et al., 2014; Hillebrand et al., 2016). If signal X has a causal influence on signal Y, then the probability density of signal Y in future conditioned on the past of X and Y is larger than the probability density of Y in future conditioned only on its own past, i.e., the past X can reduce the uncertainty of future Y.

For the selected 10-s segments, the middle two 4.096 s epochs were further selected for analysis. These time series were filtered into four frequency bands: 1–5, 5–10, 10–20, and 20–45 Hz. Then, the Hilbert transform was applied to the filtered epochs to generate instantaneous phases. For each electrode pair and frequency band, dPTE was estimated. The details of dPTE can be found in literature (Hillebrand et al., 2016). The value of dPTE_i, j_ ranges between −0.5 and 0.5. When dPTE_i, j_ > 0, the information tends to flow from time series i to time series j. When dPTE_i, j_ < 0, the information tends to flow from time series j to time series i. When dPTE_i, j_ = 0, there is no preferential direction of information flow.

#### Statistical analyses

In order to test the differences in the power spectra, three-way repeated measures analysis of variance (ANOVA) was used for within-subject variables: state, brain region, and frequency. Simple or simple-simple effects were further tested when the interactions reached the significance level. To estimate the effect size of ANOVAs, the partial η^2^ was introduced whose values of 0.2, 0.5, and 0.8 indicate a small, medium, and large effect sizes, respectively (Cohen, 1992). The *post-hoc* test was conducted using paired samples *t*-tests. Probability values were Bonferroni-adjusted for multiple comparisons, and *p* < 0.05 was set as the significance level.

For each combination of frequency band, vigilance state, and rat, the dPTE (information flow) matrices were first averaged over 60 epochs, yielding preferential information flow matrix per rat. These matrices were then averaged over rats. The average preferential information flow of each region was subsequently computed. To test the significance of the average information flow between two selected areas, randomization testing was used. Each average information flow matrix was randomly permuted across region pairs for 5,000 times. Then, we averaged the values within selected matrix areas, which formed a distribution of surrogate values to test the observed value of average information flow between two areas (*p* < 0.05). The average values of the selected areas were normalized by the maximum value of their average information flow matrix.

## Results

### EEG power spectra across states and brain regions

Three-way repeated measures ANOVA revealed significant main effects of the factor “state” [*F*_(2, 18)_ = 193.731; *p* < 0.001, partial η^2^ = 0.956], the factor “brain region” [*F*_(3.047, 27.425)_ = 77.340; *p* < 0.001, partial η^2^ = 0.896], and the factor “frequency” [*F*_(2.996, 26.964)_ = 4611.868; *p* < 0.001, partial η^2^ = 0.998]. In addition, significant interactive effects were also found for “state ^*^ brain area” [*F*_(4.365, 39.283)_ = 60.287; *p* < 0.001, partial η^2^ = 0.870], “state ^*^ frequency” [*F*_(4.878, 43.905)_ = 625.375; *p* < 0.001, partial η^2^ = 0.986], “brain area ^*^ frequency” [*F*_(4.676, 42.084)_ = 40.847; *p* < 0.001, partial η^2^ = 0.819], and “state ^*^ brain area ^*^ frequency” [*F*_(6.930, 62.373)_ = 53.493; *p* < 0.001, partial η^2^ = 0.856]. As the three interactions reached the significant level, the simple-simple effect analysis was performed.

**Figure 2:**
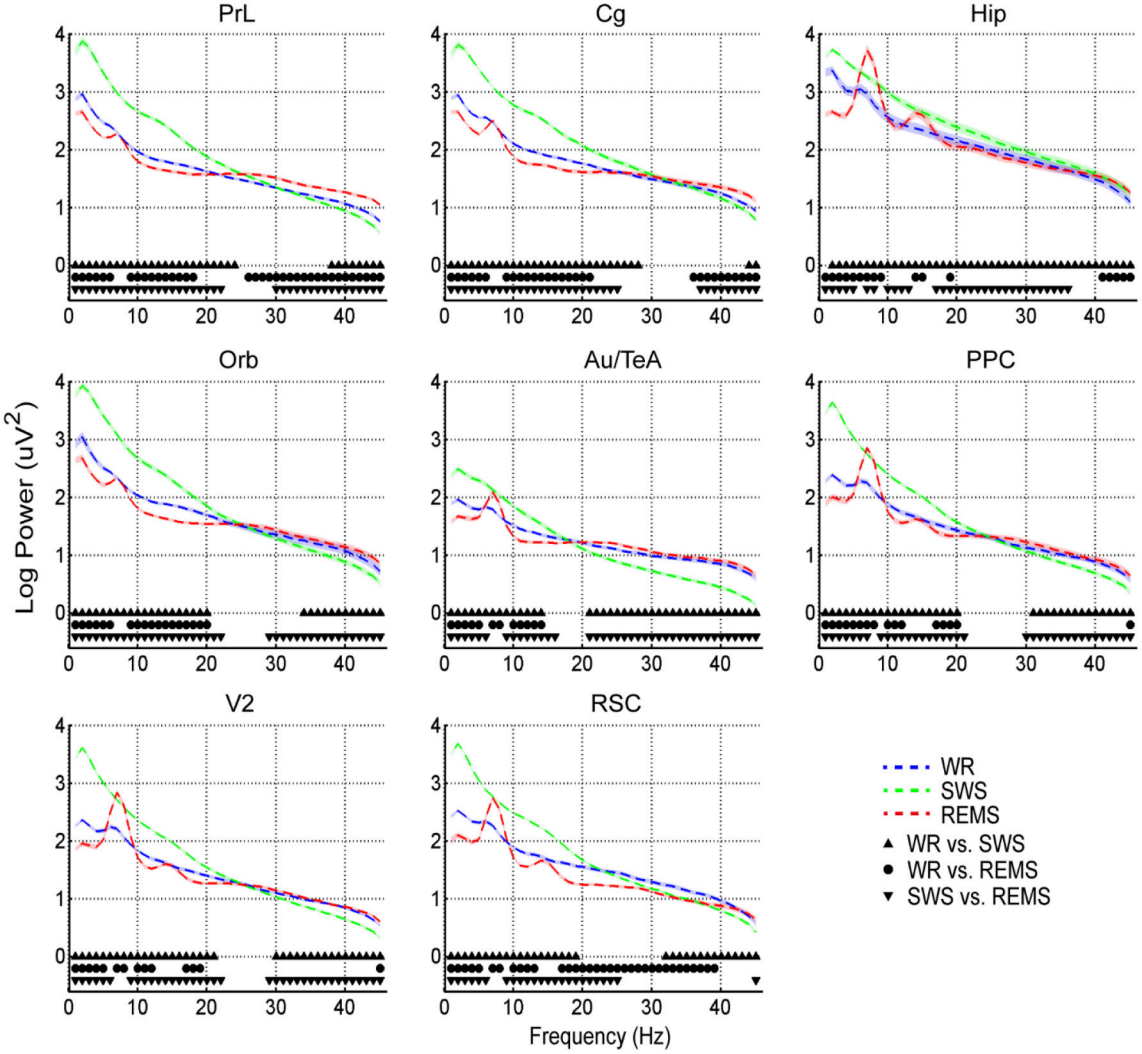
**Means (dashed lines) and standard errors (shaded areas) of log-transformed EEG power spectra during WR, SWS, and REMS across brain areas**. Solid symbols indicate the significant differences between states (*p* < 0.05, corrected).

*Post-hoc* tests indicated that power spectra during SWS were significantly higher than those during WR and REMS in the relatively low frequency oscillations (lower than around 20 Hz) across brain areas except Hip, of which significances had a wider distribution (power spectra in some regions with frequency around 8 Hz during REMS did not reach the significance level compared with those of SWS); on the contrary, SWS showed significantly lower power than WR and REMS in the relatively higher frequency oscillations (higher than around 20 Hz) (Figure 2). Interestingly, for power spectra of the low frequency oscillations, REMS had a lower power except at the frequencies around 8 Hz and its second harmonic compared with WR across all the brain areas; however, power spectra were quite different across brain areas in the higher frequency oscillations. For example, REMS showed a higher power in PrL, Cg, and Hip compared with WR in the gamma band, while there was no difference between the two vigilance states in Orb, Au/TeA, PPC, and V2 in this band (except in PPC and V2 at 45 Hz) (Figure 2). As for RSC, REMS showed a persistently lower power compared with WR from 17 to 39 Hz (Figure 2).

### Information flow across states and frequency bands

**Figure 3:**
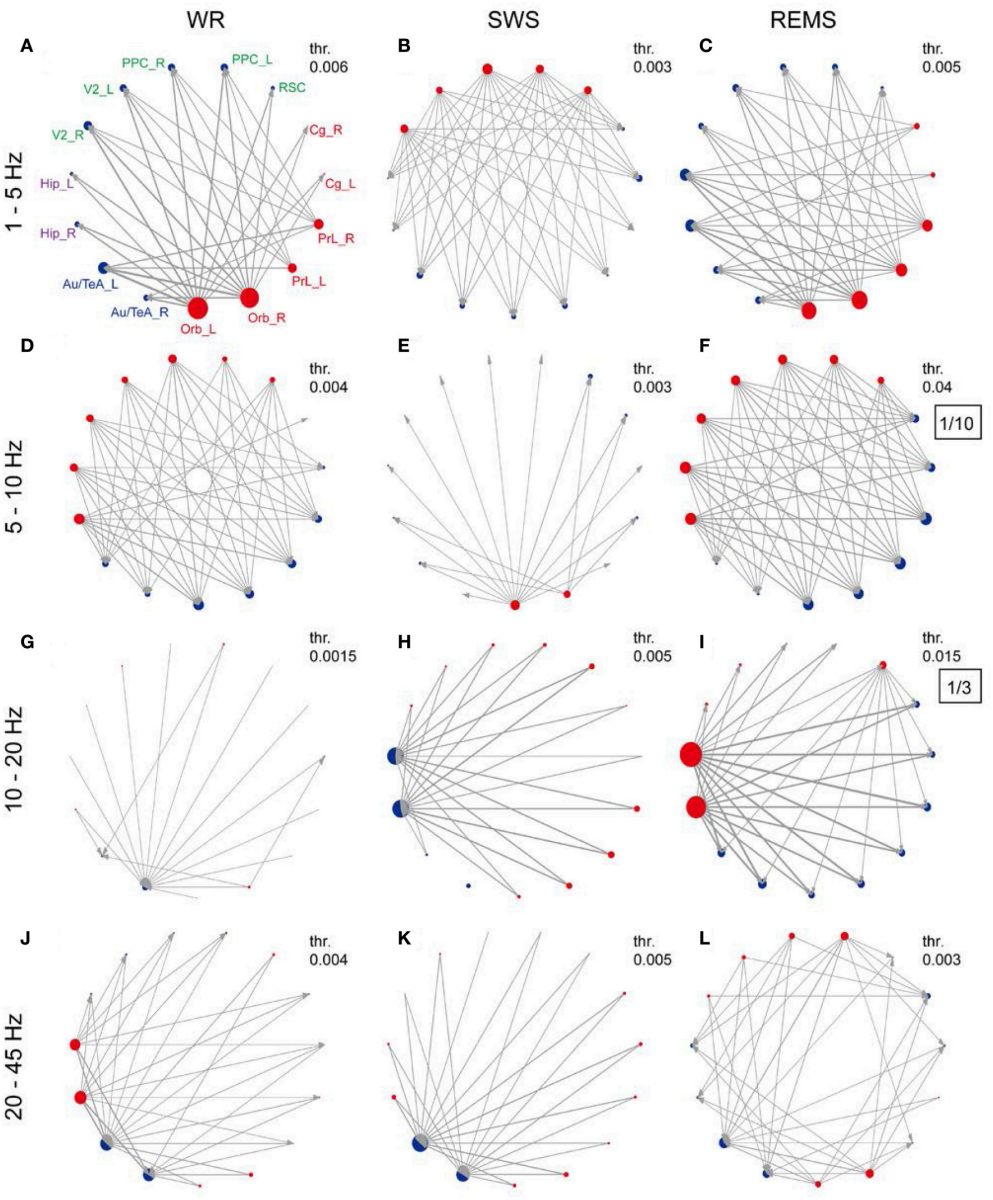
**Preferred direction of information flow for DMN regions across vigilance states and frequency bands. (A–L)** Network topology of each frequency band under each state. Red and blue dots indicate the preferential outgoing and incoming information flows of each brain region, and the node size reflects the strength of information flow (refer to Supplementary Figure 2). Line thickness and arrows indicate the strength of information flow (with thresholds in their top right corner) and the preferred direction between brain regions, respectively (refer to Supplementary Figure 3). Thresholds were chosen to highlight the relatively stronger information flow. In order to get better contrast, the nodes were grouped into prefrontal regions, parietal regions, hippocampal regions, and temporal regions, corresponding to red, green, purple, and blue abbreviations, respectively. For **(F,I)**, the values are too large, so the values were zoomed out, and the zoom ratio is shown in the black box.

The preferential direction of information flow within rat DMN was estimated using the dPTE. In the group level (10 rats), the average dPTE of each region across states and frequency bands was first computed, resulting in a preferential direction of the information flow for each region (Supplementary Figure 2). The preferential information flow of region pairs was subsequently examined (Supplementary Figure 3). Figure 3 summarizes the preferential information flow of each region and preferential information flow between them across three states with four frequency bands. Preferential information flow in the band of 1–5 Hz showed patterns indicating that prefrontal regions leaded the parietal and hippocampal regions during both WR and REMS (Figures 3A,C). Opposite patterns were observed in the band of 5–10 Hz during both WR and REMS (Figures 3D,F). More importantly, most of the information senders in 1–5 Hz were also the receivers in 5–10 Hz, and vice versa, which showed a reciprocal pattern. However, this reciprocal pattern was not found during SWS (Figures 3B,E). Additionally, in the band of 20–45 Hz, Hip and Au/TeA showed widespread outgoing and incoming information flows during WR, respectively (Figure 3J). In the band of 10–20 Hz, Hip showed a widespread incoming information flow during SWS (Figure 3H), while the opposite pattern was observed during REMS (Figure 3I). As for the Au/TeA during SWS, widespread incoming information flow was observed in the band of 20–45 Hz (Figure 3K). According to the spatial distribution of these brain regions (Figure 1, left), we could divide rat DMN into an anterior part (prefrontal regions) and a posterior part (parietal regions, Hip, and Au/TeA). Thus, the opposite patterns in WR and REMS could be described as the anterior-to-posterior pattern and posterior-to-anterior pattern, respectively.

**Figure 4:**
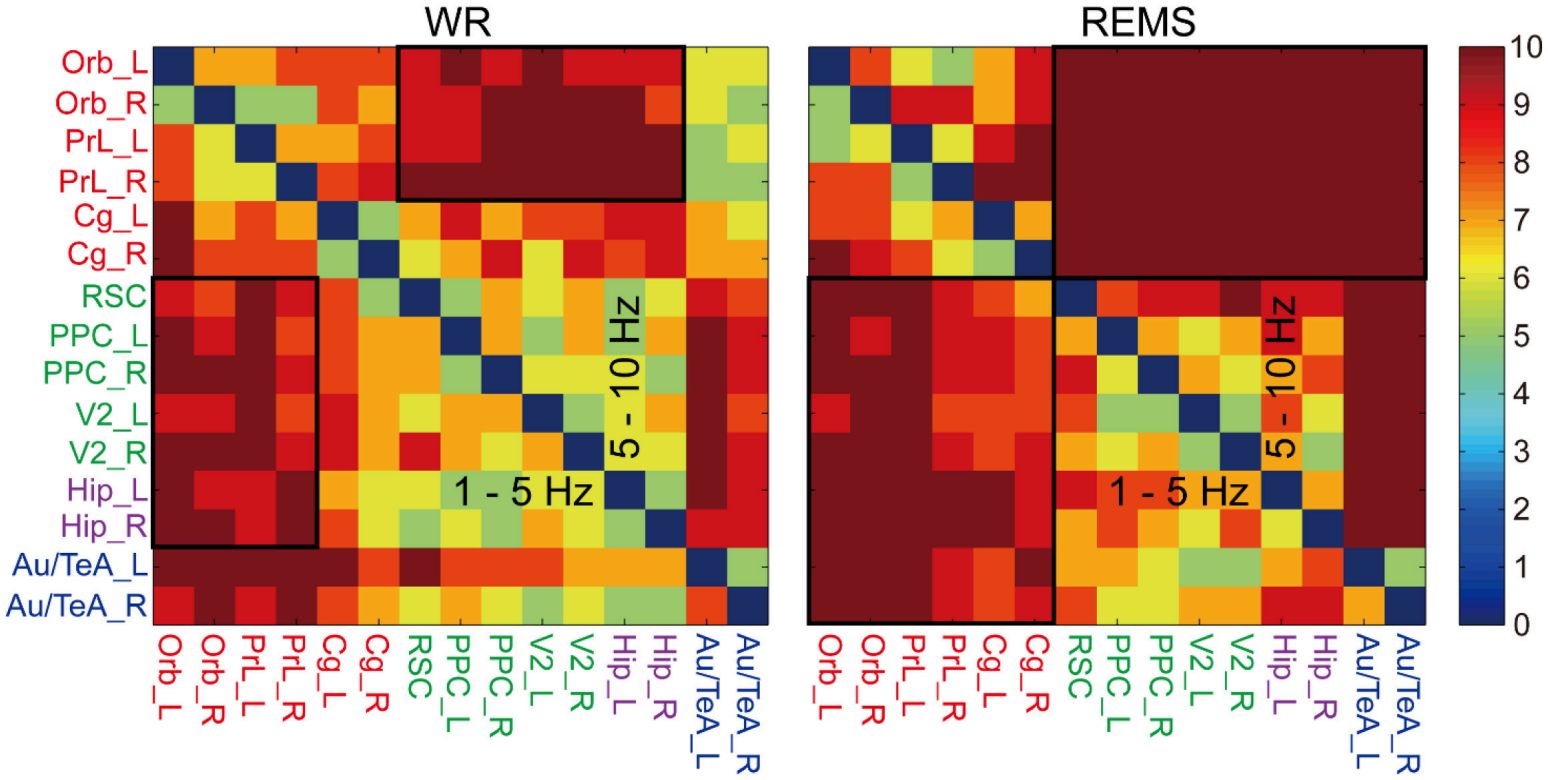
**Consistency of the preferential information flow across rats during WR and REMS (refer to Supplementary Figure 4)**. Color bar indicates the rat numbers of consistent information flow between region pairs. Black boxes show the reciprocal region pairs with relatively high consistency (the lower triangles of 1–5 and 5–10 Hz), which were selected for further randomization test. Colored abbreviations represent the grouped brain regions as before.

To further test the consistency of the preferred information flow, we also examined it across each rat. As shown in Figure 4, in the bands of 1–5 and 5–10 Hz, the reciprocal region pairs in WR and REMS showed a relatively high consistency, reflecting the stability of this pattern. For WR, the consistently reciprocal pattern included region pairs between Orb/PrL and Parietal/Hip. As for REMS, the consistently reciprocal pattern included region pairs between Orb/PrL/Cg and Parietal/Hip/(Au/TeA). Additionally, relatively high consistency was also found in region pairs related to Hip and Au/TeA (Supplementary Figure 4). Specifically, during WR, relatively high consistency of Hip-the other regions and Au/TeA-the other regions was observed in the band of 20–45 Hz; during SWS and REMS, relatively high consistency of Hip-the other regions was observed in the band of 10–20 Hz; and during SWS, relatively high consistency of Au/TeA-the other regions was observed in the band of 20–45 Hz.

Based on the high consistency, preferential information flow between areas (box areas in Supplementary Figure 4) was further tested with a randomization method. The average dPTE of region pairs ranged from −0.81 to 0.79 (normalized value), and all of them were significant [compared with a distribution of surrogate data (5,000 times), *p* < 0.001; Table 3]. To further test the opposite pattern between 1–5 and 5–10 Hz across vigilance states, Pearson correlation between upper triangles of the dPTE matrices (Supplementary Figure 3) was performed. Significantly negative correlation was found in WR and REMS (*r* = −0.62 and −0.85, respectively, *p* < 0.001). As expected, we did not find a significantly negative correlation in SWS (*r* = −0.10, *p* = 0.31).

**Table 3:** **Preferential information flow between areas**.

**State**	**Frequency band**	**Information flow**		**Normalized dPTE**	***p*****-values**
		**Out region**	**In region**		
WR	1–5 Hz	Orb, PrL	RSC, PPC, V2, Hip	0.67	*p* < 0.001
	5–10 Hz	RSC, PPC, V2, Hip	Orb, PrL	−0.76	*p* < 0.001
	20–45 Hz	Hip	All other regions	0.52	*p* < 0.001
	20–45 Hz	All other regions	Au/TeA	−0.56	*p* < 0.001
SWS	10–20 Hz	All other regions	Hip	−0.80	*p* < 0.001
	20–45 Hz	All other regions	Au/TeA	−0.81	*p* < 0.001
REMS	1–5 Hz	Orb, PrL, Cg	RSC, PPC, V2, Hip, Au/TeA	0.65	*p* < 0.001
	5–10 Hz	RSC, PPC, V2, Hip, Au/TeA	Orb, PrL, Cg	−0.72	*p* < 0.001
	10–20 Hz	Hip	All other regions	0.79	*p* < 0.001

Finally, we tested reproducibility of the dPTE matrices in different data sizes. The average dPTE matrices were generated from a randomly selected dataset of 10 rats, which consisted of 10–50 matrices per rat (i.e., 10, 20, 30, 40, and 50, each random selection was repeated 100 times). Then, we computed the correlation coefficient between the new average dPTE matrix of each state with different frequency bands and the corresponding results obtained in the main analyses above. The similarities were monotonically increased for all states with various frequency bands (Supplementary Figure 5). These results indicate similar dPTE matrices across rats.

## Discussions

Accumulating evidence indicates that local metabolic activity is tightly correlated with electrophysiological gamma power in humans (Miller et al., 2009; Jerbi et al., 2010; Ossandón et al., 2011), monkeys (Hayden et al., 2009; Hutchison et al., 2015), and rats (Thompson et al., 2013; Li et al., 2014). In the present work, we observed significantly decreased gamma power during SWS compared with that of WR across most of the regions. More interestingly, during RMES, limbic structure (PrL, Cg, and Hip) showed an increased gamma power compared to that of WR. In contrast, RSC showed a decreased gamma power. These regions with changed gamma power are in line with the metabolic variations of the human counterparts across vigilance states (Maquet et al., 1996, 1997; Braun et al., 1997; Nofzinger et al., 1997; Maquet, 2000; Fox et al., 2013; Nir et al., 2013). These increased activities in limbic regions during REMS may facilitate spatial and/or emotional memory consolidation (Popa et al., 2010; Walker, 2010; Soto-Rodriguez et al., 2016), or even be related to enhanced emotional activity in dream experience (Hobson and Pace-Schott, 2002; Fox et al., 2013). RSC has extensive connections with neocortex and archicortex areas (Vann et al., 2009), supporting sensory-cognitive activities that include multimode sensory information processing, spatial and contextual learning, and episodic memory (Robinson et al., 2011, 2014; Bucci and Robinson, 2014; Wang et al., 2016). Based on these functions, if the rat also has similar dream activity, the decreased activity in RSC during REMS may be related to the chaotic dream components, i.e., spatial-temporal bizarreness of dreams (Corsi-Cabrera et al., 2003; Hobson, 2009).

A recent modeling study suggests that the spatial patterns of resting-state networks (including DMN) in human brains can emerge only when the brain dynamics are poised around a so-called critical point (Haimovici et al., 2013). When the brain is around this critical point, the power spectra for both field potentials and fMRI signals are in a power-law fashion, and their power-law exponents change with the state of the brain dynamics (He et al., 2010; He, 2011). Thus, the increased power of slow oscillation accompanied with decreased power of high frequency oscillation during SWS in the present work (changed power-law exponent) may affect the functional coupling among brain regions (He, 2011); this possibly corresponds to the weakened coupling within DMN during SWS (Horovitz et al., 2009; Sämann et al., 2011; Chow et al., 2013). These observations provide electrophysiological evidence that the rat has a DMN which is similar to that in humans.

Besides the local activities that are similar to those in humans, we have also demonstrated a dominant anterior-to-posterior pattern of information flow in the delta band (1–5 Hz) during WR. In contrast, a pattern of posterior-to-anterior flow was also found in the theta band (5–10 Hz) during WR, involving mainly regions of the anterior-to-posterior pattern, in which the strong information senders in the delta band were also often the information receivers in the theta band. In spite of the differences in frequency bands, these results are in agreement with findings in humans (Hillebrand et al., 2016). Interestingly, this pattern was observed only in WR and REMS, but not SWS (Figure 3), suggesting a functional frequency-specific loop of information flow specific to both WR and REMS.

In humans, conscious experiences (in the sense of subjective awareness) exist in both WR and REMS (internal awareness, i.e., dream activity), but are highly suppressed during SWS (Hobson and Pace-Schott, 2002; Hobson, 2009; Nir et al., 2013). Consciousness is suggested to arise out of and depend on functional relationships among brain regions (Raichle and Snyder, 2009). Recent sleep studies also support this notion by showing a decreased functional integration of the brain during non-rapid eye movement (NREM) sleep compared with WR (Spoormaker et al., 2010; Larson-Prior et al., 2011; Boly et al., 2012; Tagliazucchi et al., 2013; Uehara et al., 2014). Furthermore, compared with the other brain networks, the information integration of DMN may play a pivotal role in maintaining consciousness (Uehara et al., 2014). DMN showed a highly disrupted integrity during SWS (Horovitz et al., 2009; Larson-Prior et al., 2011; Sämann et al., 2011; Chow et al., 2013), which was recovered during REMS (Chow et al., 2013). Based on the highly evolved corresponding structures (Hobson, 2009) and dream like behavior activity (Yu et al., 2015) in rats, they may also have similar conscious activity (dream activity) during REMS. Thus, the frequency-specific loop of information flow in rat DMN may reflect, and perhaps even indicate, conscious experience.

Prefrontal cortex can integrate multimodal sensory and limbic information (Nauta, 1971; Groenewegen and Uylings, 2000; Öngür and Price, 2000). Recent data demonstrated that low-frequency rhythmically modulates the excitability of local neuronal ensembles (Buzsáki, 2006), in such a way of cross-frequency coupling that the phase of delta oscillation modulates the amplitude of theta oscillation, and the theta phase further modulates the gamma amplitude, forming a hierarchical control of neuronal excitability (Lakatos et al., 2005; Buzsáki, 2006; Schroeder and Lakatos, 2009). Therefore, the prefrontal-to-parietal/Hip delta connectivity may provide a top-down modulation of local excitability linked to information processing. Moreover, recent observations suggest that delta-band oscillations can provide an instrument for attentional selection by phasic regulation of neuronal excitability (Lakatos et al., 2008), and the phase of delta (2–4 Hz) can predict the multiunit activity response (Whittingstall and Logothetis, 2009); these findings further support this idea. This top-down modulation may be a fundamental mechanism of dynamic brain organization, supporting conscious awareness in an energy-efficient way (the result of low bandwidth) (Raichle and Snyder, 2009).

Temporal binding of multiregional information is important for conscious awareness (Damasio, 1989; Buzsáki, 2006). Neural oscillations can establish precise temporal binds among distributed neuronal responses. More specifically, high frequency (beta and gamma) coordinates local cortical integration and low frequency (theta and alpha) preferentially coordinates long-range integration (Uhlhaas and Singer, 2010; Buzsáki et al., 2013). Given that DMN is a set of widespread long-range brain regions (Lu et al., 2012, 2016), we reason that parietal/Hip-to-prefrontal theta band information flow is well suited to bind information of the parietal/Hip areas in precise temporal relations (bottom-up integration). This idea is consistent with the proposition that theta phase coding is important in long-range communication (Lisman and Jensen, 2013). Accordingly, the observation of the mirrored information flow matches the conception of reentry (including “top-down” and “bottom-up”), which may support consciousness as a mechanism for functional integration in the brain (Edelman and Gally, 2013).

Compared with that of WR, parietal/Hip-to-prefrontal theta band information flow was stronger (orders of magnitude) during REMS, and Au/TeA and Cg were also engaged in the loop (Supplementary Figure 3, Figure 4). This may reflect intensified conscious awareness (Fox et al., 2013) and enhanced emotional activities during REMS (Hobson and Pace-Schott, 2002; Fox et al., 2013). Coordinated interactions between spindles and ripples play a crucial role in the off-line consolidation of memory during SWS (Siapas and Wilson, 1998; Mölle et al., 2009). Therefore, the cortico-hippocampal propagation in the band of spindle (10–20 Hz) may be associated with the modulation of hippocampal sharp-wave ripple (SWR) activities (Sirota et al., 2003), which transmit information from Hip to the neocortex during SWS (Chrobak and Buzsáki, 1994; Stickgold, 1998). On the contrary, during REMS, the pattern of information flow is reversed, i.e., from neocortex to Hip (Chrobak and Buzsáki, 1994; Stickgold, 1998). Thus, the hippocampo-cotrical propagation in the band of 10–20 Hz may be the same modulation in the reverse direction. Moreover, the hippocampo-cotrical direction of information transmission also happens during WR (Chrobak and Buzsáki, 1994; Stickgold, 1998). In the current work, the hippocampo-cotrical propagation in the low-gamma band (20–45 Hz) during WR may be involved in the function that the hippocampo-cotrical low-gamma activity coordinates the neocortical response to hippocampal SWR (Remondes and Wilson, 2015). There is no clear explanation for the low-gamma information flow from the other DMN areas to Au/TeA during both WR and SWS; future task-based studies may contribute to solving this problem.

Although the local activities and information flows within electrophysiological rat DMN are similar to those in human DMN, there are still some differences. Specifically, effective connectivity studies based on human resting-state fMRI highlight that the posterior cingulate cortex (PCC, corresponding to RSC in rat) is a driven hub within DMN (Jiao et al., 2011; Zhou et al., 2011; Di and Biswal, 2014). In contrast, we have not found that the RSC acts as a hub in rat DMN. Furthermore, as mentioned above, the frequency bands of the frequency-specific loop of information flow are different from that in humans (Hillebrand et al., 2016). These differences may reflect a species-specific difference: rat DMN is different from that of humans in that it further includes Orb and the entire cingulate cortex (Cg and RSC) (Lu et al., 2012); furthermore, compared with the hub region located in human PCC, the most prominent hub of the DMN is the prefrontal/cingulate regions in rodents (Gozzi and Schwarz, 2016).

In conclusion, using the electrophysiological method, we have found that the changed gamma power within rat DMN is consistent with the metabolic variations of the homologous human regions across vigilance states, further supporting the similarity of DMN between rats and humans. Interestingly, we also found cross-frequency interaction between anterior-posterior part of DMN during WR and REMS, but not SWS. These cross-frequency interactions may reflect a reentry loop of information flow, reflecting conscious awareness during both WR and REMS.

## Author contributions

YX, DY, and WJ conceived and designed the study. WJ, YZ, and FG conducted the experiments. WJ and DG performed initial analysis and prepared draft manuscript. WJ, PV, and DY reviewed data interpretation. YX edited and approved final manuscript.

## Funding

This work was funded by the National Natural Science Foundation of China (Grant No. 81371636, 61527815, 81571770, 81330032), Special-Funded Program on National Key Scientific Instruments and Equipment Development of China (No. 2013YQ490859), the 111 project B12027.

### Conflict of interest statement

The authors declare that the research was conducted in the absence of any commercial or financial relationships that could be construed as a potential conflict of interest.
