# Supplementary material for: Reentrant Information Flow in Electrophysiological Rat Default Mode Network
Source: Front Neurosci. 2017 Feb 27;11:93. doi: 10.3389/fnins.2017.00093 (PMC5326791; doi:10.3389/fnins.2017.00093)
Supplement: Supplementary file 1 [file DataSheet1.DOCX]

Supplementary Material

Reentrant information flow in electrophysiological rat default mode network

Wei Jing, Daqing Guo, Yunxiang Zhang, Fengru Guo, Pedro Antonio Valdés-Sosa, Yang Xia*, Dezhong Yao

*** Correspondence:** Yang Xia: xiayang@uestc.edu.cn

# Data selection

To ensure that the rats were sufficiently adapted to the recording environment before obtaining data, only the data of the last 24 hours were included in our analysis. Data of both slow-wave sleep (SWS) and rapid-eye-movement sleep (REMS) were selected from 8:00 to 12:00 (this period was rich in sleep activity). For each SWS or REMS segment, the state period > 100 s was included for selection. Thirty ten-second, artifact-free segments were selected in the middle (60 s) of the epochs were selected for SWS. The 12:00 to 15:00 period was excluded because of the disturbance of the experimenter (Abou-Ismail et al., 2008). If the selected data segments were less than the assumed 30 segments, the data from 15:00 to 20:00 were then included. Thirty ten-second, artifact-free segments of wakeful rest (WR) data were selected from the rest of the 17-hour daily cycle, except from 8:00-15:00.

REMS can be divided into two types (tonic and phasic REMS) both in humans and rats (Horne, 2000;Montgomery et al., 2008). But in rats, the phasic REMS is less than 5% of the total REMS (Montgomery et al., 2008). Thus, we excluded the data of phasic REMS for analysis. For each rat, ten epochs (60 s per epoch) of the REMS data were first selected, then the phasic REMS data were eliminated using the procedures reported by Mizuseki et al. (Mizuseki et al., 2011). Therefore, a total of 155.761s (2.6% of selected REM sleep data) was eliminated. At last, we selected thirty continuous ten-second segments for each rat.

# Supplementary Figures


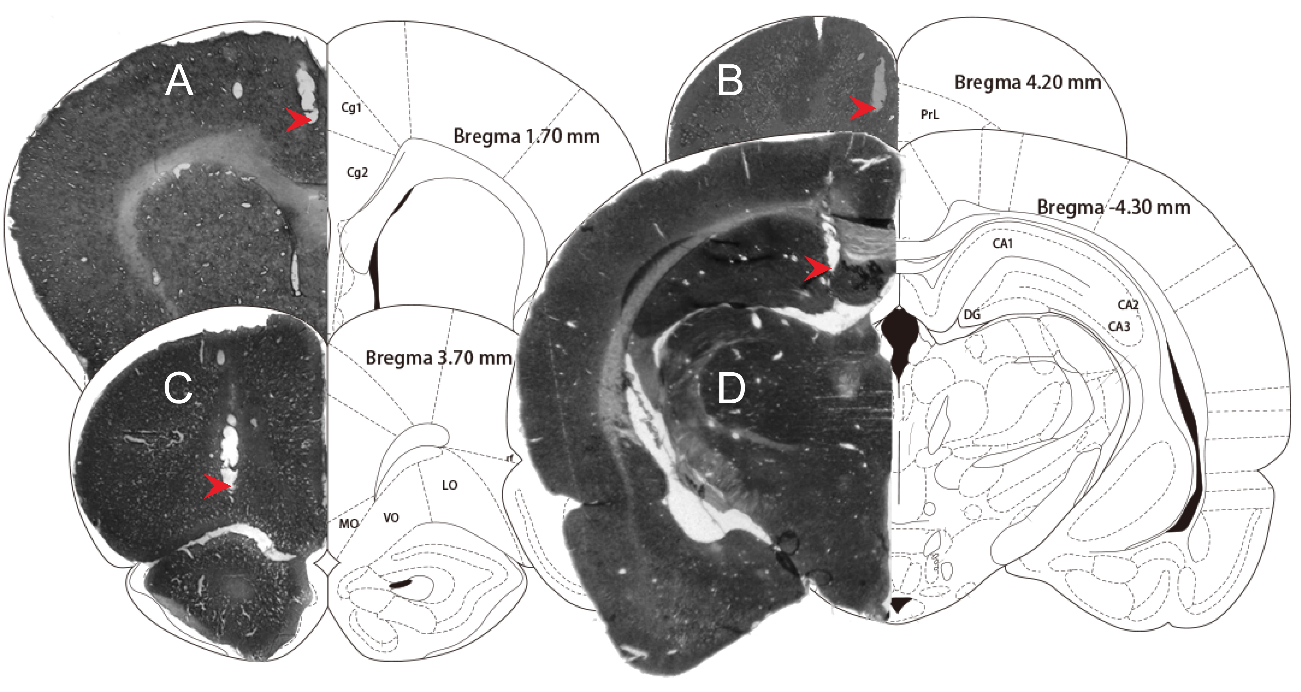


**Supplementary Figure 1.** Histological tests used to determine electrode positions [atlas adapted from (Paxinos and Watson, 2005)]. (A) Electrode position of CG. (B) Electrode position of PrL. (C) Electrode position of OFC. (D) Electrode position of Hip. The red arrows denote the recording tips.


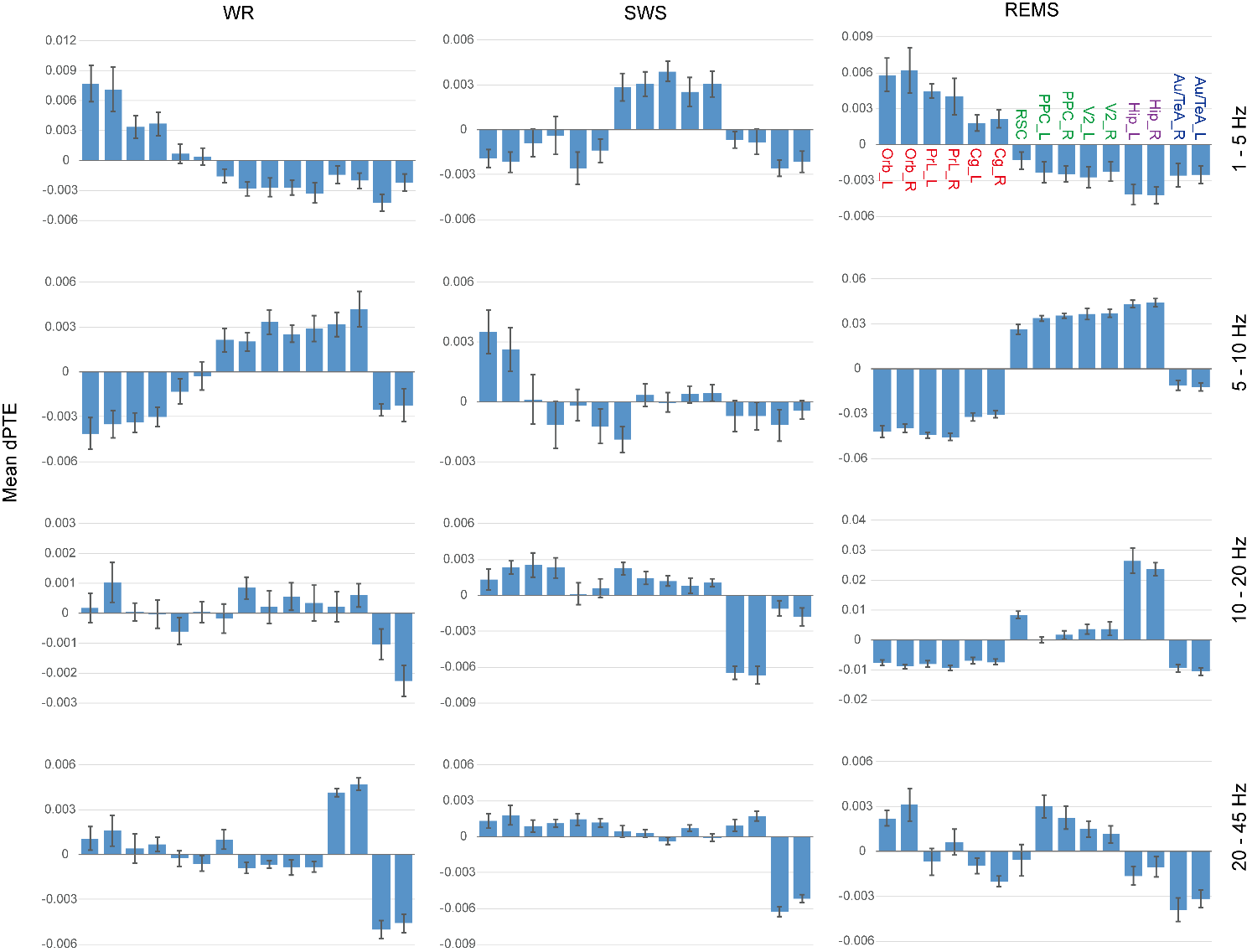


**Supplementary Figure 2.** Mean preferred direction of information flow and standard errors for each region across vigilance states and frequency bands. The regions of rat DMN are grouped into prefrontal regions, parietal regions, hippocampal regions, and temporal regions, corresponding to red, green, purple, and blue abbreviations, respectively. WR, SWS and REMS are three vigilance states.


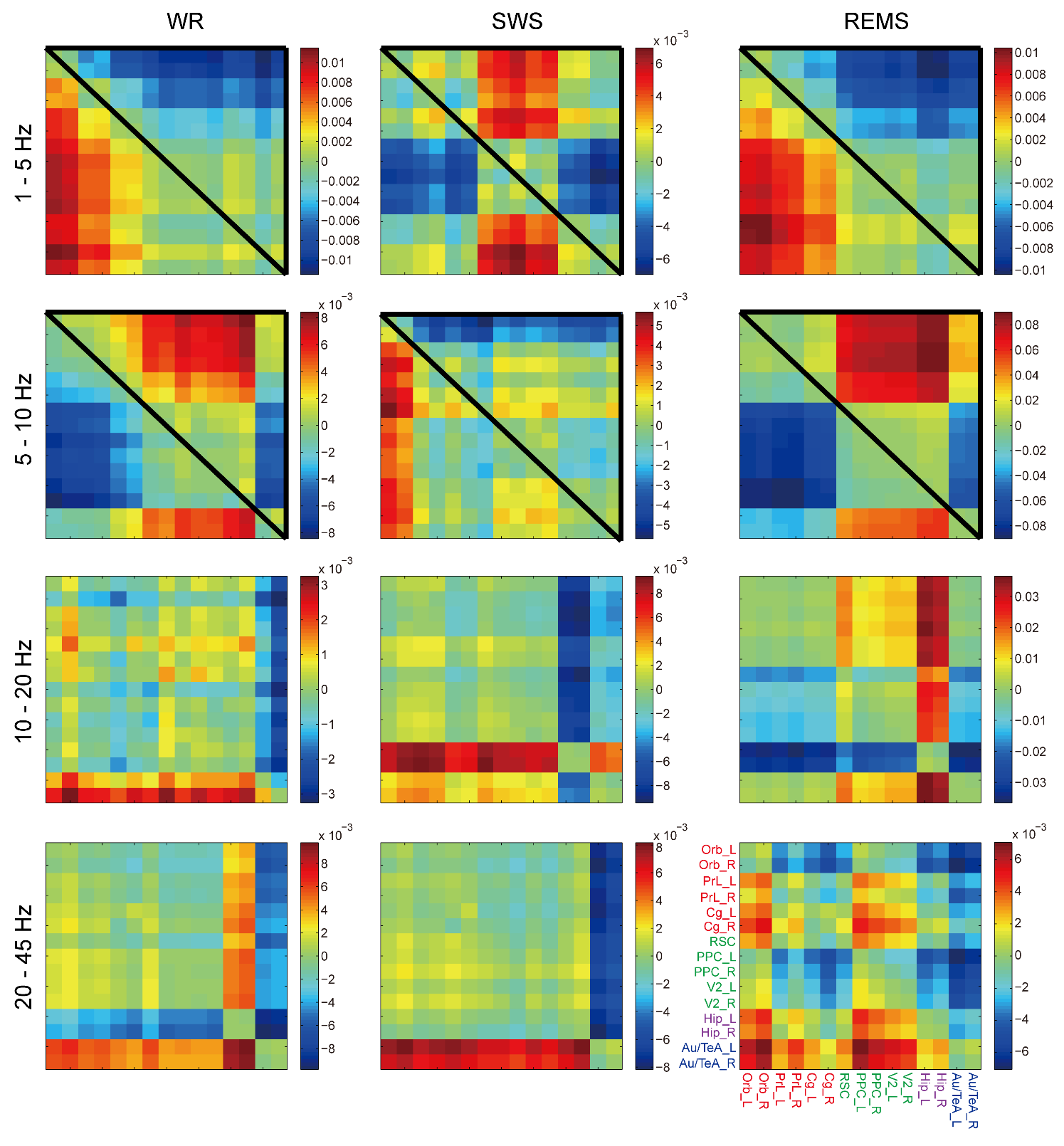


**Supplementary Figure 3.** Color-coded connectivity matrices of mean dPTE between regions across vigilance states and frequency bands. Hot and cold colors indicate information outflow and inflow (column to row), respectively. The black triangle areas were selected to further compute the correlations between 1-5 and 5-10 Hz across states. The abbreviations are the same as those in Supplementary Figure 2.


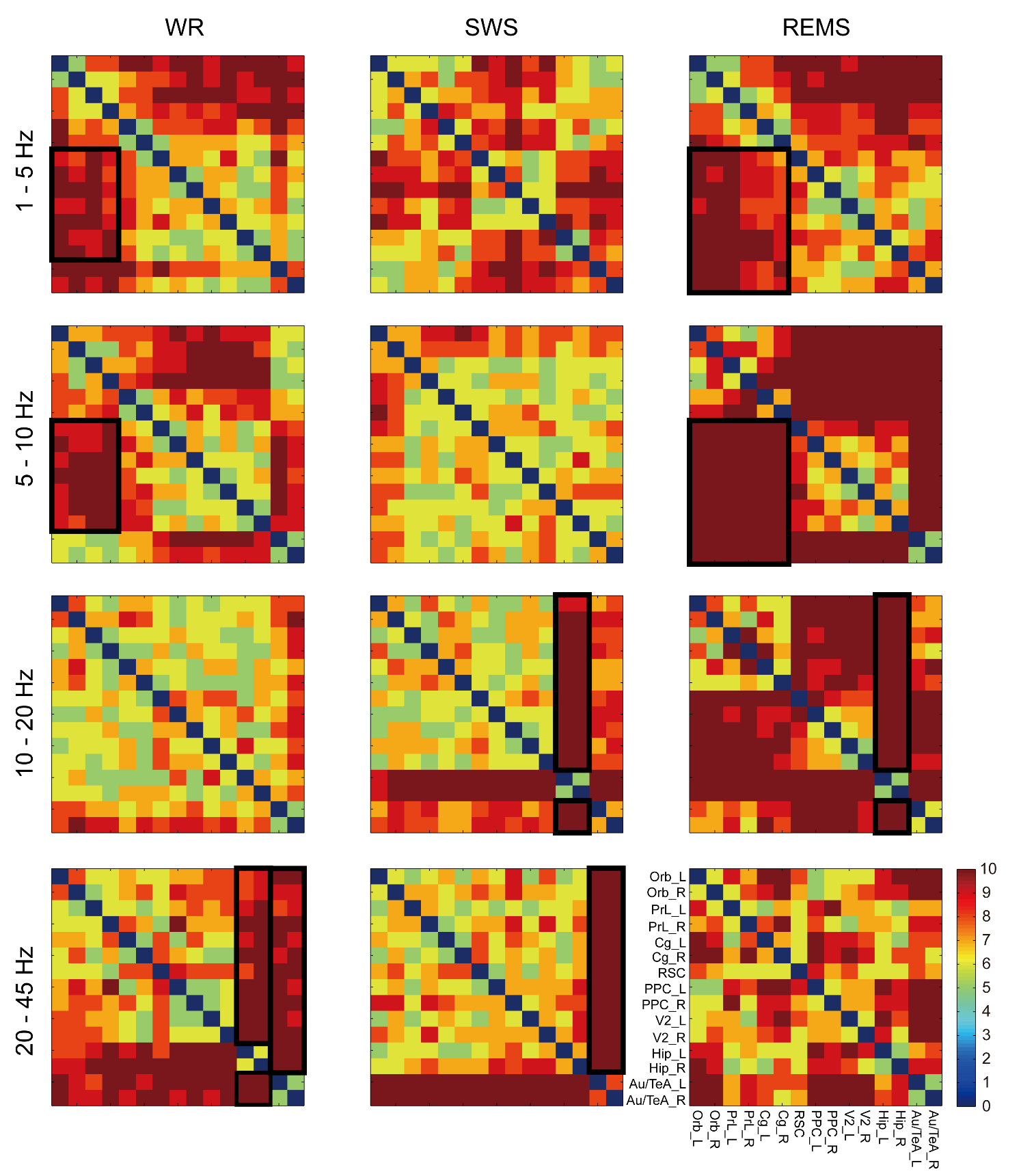


**Supplementary Figure 4.** Consistency of the preferred information flow for all frequency bands and states across rats. Color bar indicates the number of rats with consistent information flow between region pairs. Black box shows the areas with relatively high consistency and were selected to further randomization test.


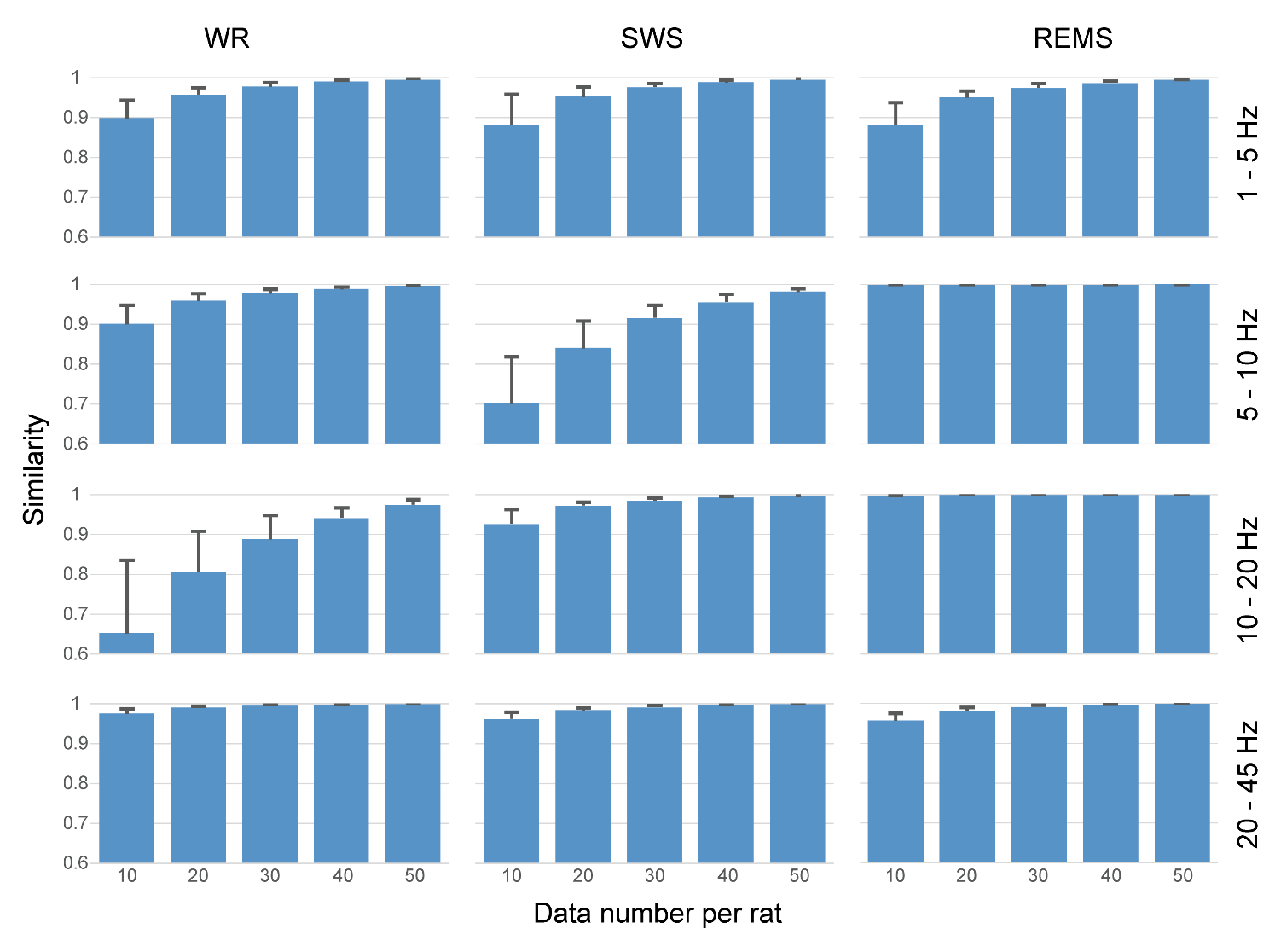


**Supplementary Figure 5.** The reproducibility of the average dPTE matrices derive from different subsets of data sizes. Correlation coefficient was calculated based on average dPTE matrices generated from randomly selected subsets consisting of 10-50 matrices per rat (each random selection was repeated 100 times, total 10 rats), which was computed between the new resultant and the one we obtained using the original 60 matrices per rat.

**References**

Abou-Ismail, U.A., Burman, O.H.P., Nicol, C.J., and Mendl, M. (2008). Let sleeping rats lie: Does the timing of husbandry procedures affect laboratory rat behaviour, physiology and welfare? *Applied Animal Behaviour Science* 111**,** 329-341.

Horne, J.A. (2000). REM sleep - by default? *Neurosci Biobehav Rev* 24**,** 777-797.

Mizuseki, K., Diba, K., Pastalkova, E., and Buzsaki, G. (2011). Hippocampal CA1 pyramidal cells form functionally distinct sublayers. *Nature Neuroscience* 14**,** 1174-U1235.

Montgomery, S.M., Sirota, A., and Buzsaki, G. (2008). Theta and gamma coordination of hippocampal networks during waking and rapid eye movement sleep. *J Neurosci* 28**,** 6731-6741.

Paxinos, G., and Watson, C. (2005). *The rat brain in stereotaxic coordinates.* Amsterdam ; Boston: Elsevier Academic Press.
